# Influence of surface topography on the human epithelial cell response to micropatterned substrates with convex and concave architectures

**DOI:** 10.1186/1754-1611-8-13

**Published:** 2014-06-19

**Authors:** Mee-Hae Kim, Yoshiko Sawada, Masahito Taya, Masahiro Kino-oka

**Affiliations:** 1 Department of Biotechnology, Graduate School of Engineering, Osaka University, 2-1 Yamadaoka, Suita, Osaka 565-0871, Japan; 2 Division of Chemical Engineering, Graduate School of Engineering Science, Osaka University, 1-3 Machikaneyama-cho, Toyonaka, Osaka 560-8531, Japan

**Keywords:** Human epithelial cells, Convex architecture, Concave architecture, Spatial cell distribution, Stereoscopic cell imaging, Cytoskeletal formation, Cell-cell interaction

## Abstract

**Background:**

Understanding the fundamental mechanisms underlying the cellular response to topographical surface features will extend our knowledge regarding the regulation of cell functions. Analyzing the cellular response to different topographical features, over multiple temporal and spatial scales, is central to understanding and guiding several biological functions. We used micropatterned substrates with convex and concave architectures to evaluate the behaviors of human epithelial cells on these substrates.

**Results:**

Pillar and pit substrates caused heterogeneous spatial growth and distribution, with differences in cell density, over 48 h. Regional densities and distribution were significantly increased at pillar sidewalls, and at pit sidewalls and bottoms compared with those on flat unpatterned areas. Time-lapse observations revealed that different mechanisms of cell migration were dependent upon pillar and pit features. Cells on pillar substrate migrated towards the sidewall, whereas cells on pit substrate tended to move towards the sidewalls and bottom. Cytoskeletal staining of F-actin and vinculin showed that this migration can be attributed to difference in spatial reorganization of actin cytoskeleton, and the formation of focal adhesions at various points on the at the convex and concave corners of pillar and pit substrates. Cells cultured on the pillar substrate had stress fibers with extended filopodia and immature focal contacts at the sidewalls and convex corners, similar to those on the flat unpatterned substrate. Cells at the sidewalls and concave corners of pit substrate had more contractile stress fibers and stable focal contacts compared with cells on the pillar substrate. We also found that the substrate structures affect cell-cell contact formation *via* E-cadherin, and that this was associated with reorganization of the actin cytoskeleton at the sidewall, and at the convex and concave corners of the substrate.

**Conclusion:**

Migration is an important factor affecting spatial growth and distribution. Heterogeneity at various locations was caused by different migratory behaviors at the convex and concave corners of pillar and pit substrates. We propose that this investigation is a valuable method for understanding cell phenotypes and the heterogeneity during spatial growth and distribution of epithelial cells during culture.

## Introduction

In tissue engineering, various efforts have been made to promote tissue regeneration. Studies have shown that providing an appropriate environment, with chemical or physical support, is important for cellular functions such as cell adhesion, proliferation, migration and differentiation [1-3]. In particular, cell adhesion on scaffold surfaces is a primary step for guiding cellular function and further tissue generation [3]. By understanding the manner in which cells interact with their physical environment, it might be possible to control cellular behavior through the fabrication of substrates with unique physical properties [4]. These approaches could allow researchers to study the dynamic responses of cells to well-defined micropatterned substrates, and the effects of modulating cell behaviors such as cell–cell and cell–substrate interactions with respect to using such constructs for tissue replacement [2,4].

Many approaches to manipulate the cell microenvironment have been conducted on micropatterned surfaces. The behaviors of many cell types have been examined using various micropatterned substrates created by a variety of microlithography and micropatterning techniques [2-16]. Of these modified surfaces, regular micropatterns affected cell responses, controlling cell morphology and function, when compared with cells grown on unpatterned flat substrates [2,3]. Green *et al.* studied the growth rate of human abdominal fibroblasts cultured on substrates patterned with square pillars or pits. They observed that cells were more sensitive to pillars or pits with smaller sizes [14]. The influence of microarchitecture on cell behavior with respect to morphology and functionality is further exemplified by the ability of cells to acutely sense variability in topographic cues. However, there have been few systematic analyses into the impact of micropatterned features on cell adhesion and migration mechanisms. Therefore, analyzing the cell responses to different topographical cues, acting over multiple temporal and spatial scales, is central to understanding and guiding several biological functions.

In this study, micropatterned substrates with convex and concave architectures were established to assess the responses of human epithelial cells to these substrates and to determine their spatial growth and distribution. We investigated the fundamental mechanisms of cell and culture surface interactions with respect to the formation of the actin cytoskeleton, focal adhesion, and cell-cell contacts.

## Materials and methods

### Fabrication of micropatterned substrates

Micropatterned substrates were provided by Kuraray Co., Ltd. (Kurashiki-shi, Japan). Two different topographic patterns, pillar and pit, were fabricated in polystyrene using a UV-lithographic technique. The fabrication process was completed by growing SiO_2_ using a vacuum deposition system on the substrates. A schematic outlining micropatterned surfaces composed of pillar and pit are shown in Additional file 1: Figure S1. The different spatial aspects for the pillar and pit features were: the top surface; the sidewalls; the bottom surface; and gaps. The gaps refer to spaces between adjacent pillar and pit. Fabricated samples were observed with a scanning electron microscope (Additional file 1: Figure S1). The dimensions of the pillar features were 50.8 ± 0.56 μm wide and 25.9 ± 0.18 μm high, with a pitch of 198.0 ± 0.57 μm. The dimensions of pit features were 53.8 ± 0.75 μm wide and 22.6 ± 0.59 μm high, with a pitch of 195.2 ± 3.48 μm. The pitch sizes were set to specific values so as to clarity an individual topography itself effects.

### Cells and culture conditions

Infinity telomerase immortalized human epithelial cells (hTERT-HME1; Clontec Laboratories, San Diego, CA, USA) were thawed and incubated in a 25-cm^2^ flask (Nunc, Roskilde, Denmark). Unless otherwise stated, the cells were cultivated in serum-free medium containing 10 μg/ml insulin (HuMedia-KG2; Kurabo Industries, Osaka, Japan) at 37°C under a 5% CO_2_ atmosphere. For experiments, the seeding density of viable cells (*X*_0_), determined by trypan blue exclusion staining, was 8.0 × 10^3^ cells/cm^2^. Culture medium was replaced every 72 h.

### Determination of spatial cell distribution

The procedures used for staining of cytoplasm and nuclei were similar to those described previously [15]. Briefly, cells were rinsed twice with phosphate-buffered saline (PBS, Sigma-Aldrich, MO, USA). Samples were incubated at 37°C under an atmosphere with 5% CO_2_ in HuMedia-KG2 containing CellTraker Green CM-FDA (Molecular Probes, Eugene, OR, USA) to stain the cytoplasm of living cells. After 45 min, the cells were further incubated for 30 min in HuMedia-KG2 without CellTraker Green CM-FDA and then were rinsed twice with PBS.

Cells were fixed with 3.7% paraformaldehyde in phosphate buffer (Wako Pure Chemical Industries, Osaka, Japan) for 10 min at room temperature and rinsed with PBS. They were then soaked in PBS with 0.25% Triton X-100 for 4 min. Cells were washed three times with PBS and then counterstained with TOPRO-3 (Molecular Probes) for visualization of nuclei. Cells were then observed using a confocal laser-scanning microscope (CLSM, model FV-300; Olympus, Tokyo, Japan) with a 60× objective lens. Two-dimensional (2-D) images were generated by scanning along the longitudinal height of the bottom surface. The resolution of each 2-D image was 256 × 256 pixels, which covered 0.25 mm^2^ of the captured area with 256 gray level, ranging from 0 (black) to 255 (white). A 2-D image was captured every 0.6 μm along the *z*-axis. The signal intensities for CellTraker Green CM-FDA and TOPRO-3 were obtained by exciting at the corresponding wavelengths of 488 and 633 nm, respectively.

**Figure 1:**
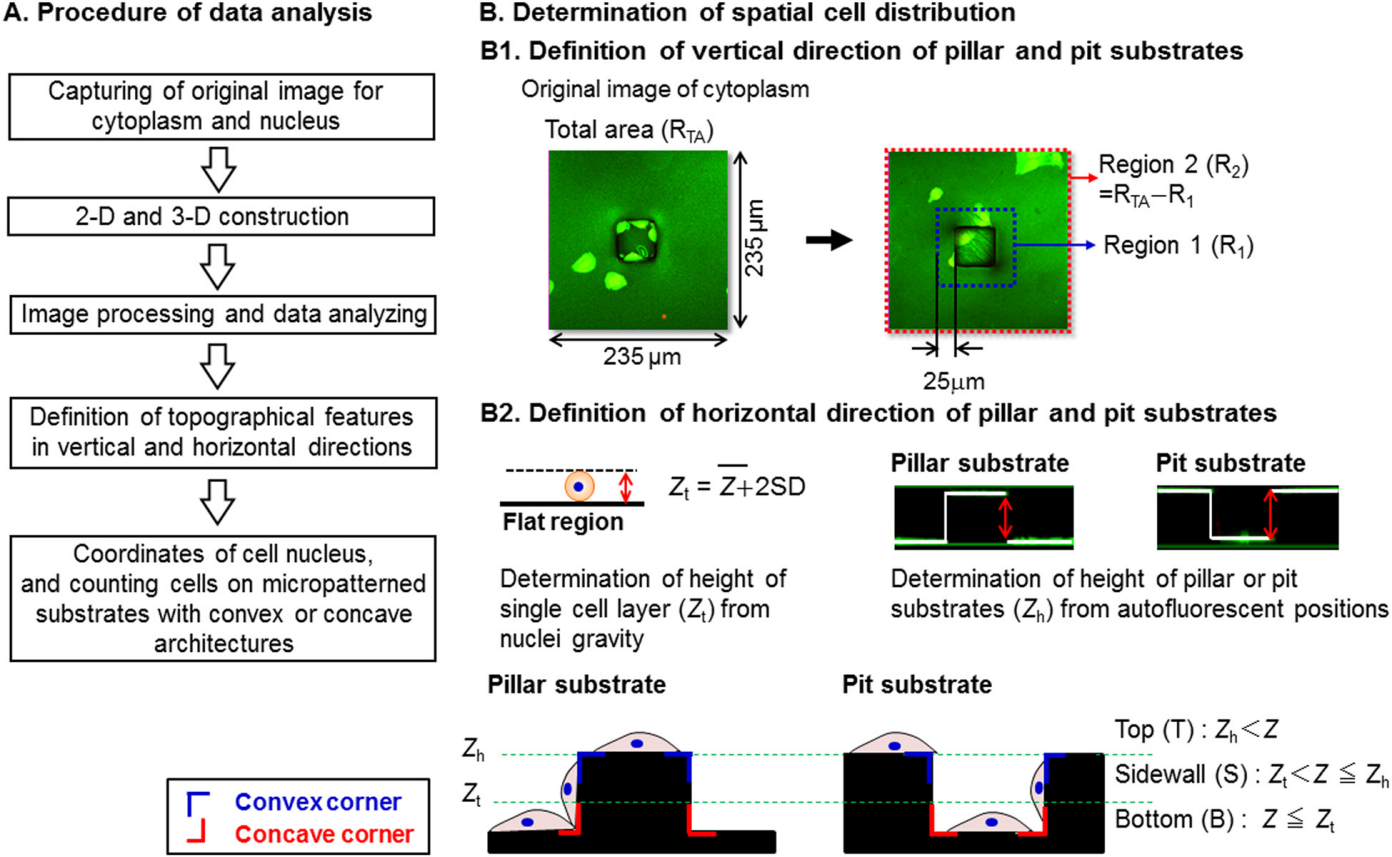
**Schematic showing the experimental procedures used for analyzing distribution of spatial cell nuclei. (A)** Whole procedure of data analysis and **(B)** determination of spatial cell distribution on micropatterned substrates with convex or concave architectures.

The experimental procedure to determine the number and spatial distribution of epithelial cells cultured on micropatterned substrates with pillars and pits is outlines in Figure 1. Spatial cell distribution was measured in the vertical and horizontal directions. To measure the cell number and distribution on micropatterned substrates in vertical direction, 2-D images were stacked and stereoscopic images were analyzed to estimate the location of cytoplasms and nuclei using Image-Pro Plus version 6.0 software (Media Cybernetics, Silver Spring, MD, USA). The region of interest (ROI; 235 μm × 235 μm) was defined as the total area (R_TA_) and was divided into two regions (R_1_ and R_2_). The densities and distributions of cells in the vertical direction were determined using ROI. We determined the number of adherent cells at 24 and 48 h and calculated the ratio (*X*_48_/*X*_24_) of adherent cells between these time points.

For reconstruction of a three-dimensional (3-D) image, the 2-D images were stacked, and the stereoscopic image was analyzed to estimate the location of nuclei using Image-Pro Plus version 6.0 software. Stereoscopic images were subjected to an algorithm with serial processes for primary noise removal, particle numbering, secondary noise removal, and 3-D construction.

To define the spatial distribution of epithelial cells on the micropatterned substrates in the horizontal direction, image capturing was conducted using epithelial cells cultured for 48 h; the coordinates for nucleus gravity were estimated. To analyze the local distribution of nuclei, it was assumed that the gravity height of nuclei on the bottom surface (*Z*) was lower than a given threshold value (*Z*_t_) as follows:

(1) $$Z_{t}=Z_{m}+\;2\mathrm{SD}$$

where the average height of nucleus gravity (*Z*_m_ = 4.5 μm) and standard deviation (SD = 1.2 μm) were determined in advance for cells cultured on the micropatterned substrates. The position of the bottom surface (*Z* = 0) was determined by measuring the autofluorescence of the culture vessel. Additionally, the height of the surface (*Z*_h_) was determined by measuring autofluorescence at the top and bottom of the surface (*Z*_h_ = 20 μm). The distribution of nuclei on the micropatterned substrate at 24 h can be seen in Figure 1. The distribution of nuclei can be compartmentalized to three topographic regions: the top (T; Z_h_ < Z); the sidewall (S; Z_t_ < Z ≤ Z_t_); and the bottom (B; Z ≤ Z_t_). The spatial growth and distribution of cell nuclei on micropatterned substrates were determined for the top, sidewall and bottom of pillar and pit substrates.

### Observation of cell behaviors

Time-lapse observation for dynamic behavior was conducted by obtaining images every 10 min at several positions using a custom-made observation tool [16]. Time-lapse images of cells at 48 h were traced backward and their dynamic movements analyzed along with division and migratory processes. Growth ability was estimated by comparing the number of dividing cells (*R*_d_) with the total number of cells at 48 h after seeding.

### Immunofluorescence staining

The procedure for immunofluorescence staining was similar to that described previously [17]. Cells were fixed with 3.7% paraformaldehyde in phosphate buffer for 10 min at room temperature and rinsed with PBS. They were then soaked in PBS with 0.25% Triton X-100 for 4 min. After masking of non-specific proteins by incubation in Block Ace (Dainippon Sumitomo Pharma Co., Ltd., Osaka, Japan) for 1 h at ambient temperature, cells were treated with a primary antibody at 4°C overnight. The primary antibodies used were against vinculin (Santa Cruz Biotechnology, CA, USA) or anti-E-cadherin (Santa Cruz Biotechnology) and were diluted appropriately in PBS containing 10% Block Ace. Cells were washed with Tris-buffered saline and then incubated with Alexa Fluor 594-conjugated goat anti-mouse IgG (Molecular Probes) for 1 h. F-actin was stained with Alexa Fluor 488 phalloidin (Molecular Probes). Cells were observed using a CLSM with a 60× objective lens.

### Statistical analysis

All experiments were conducted at least three times and data were expressed as means with standard deviations. Student’s *t*-test was used to determine the statistical significance among data sets. A *p*-value less than 0.05 was considered significant.

## Results

### Spatial growth and distribution of epithelial cells on micropatterned substrates

**Figure 2:**
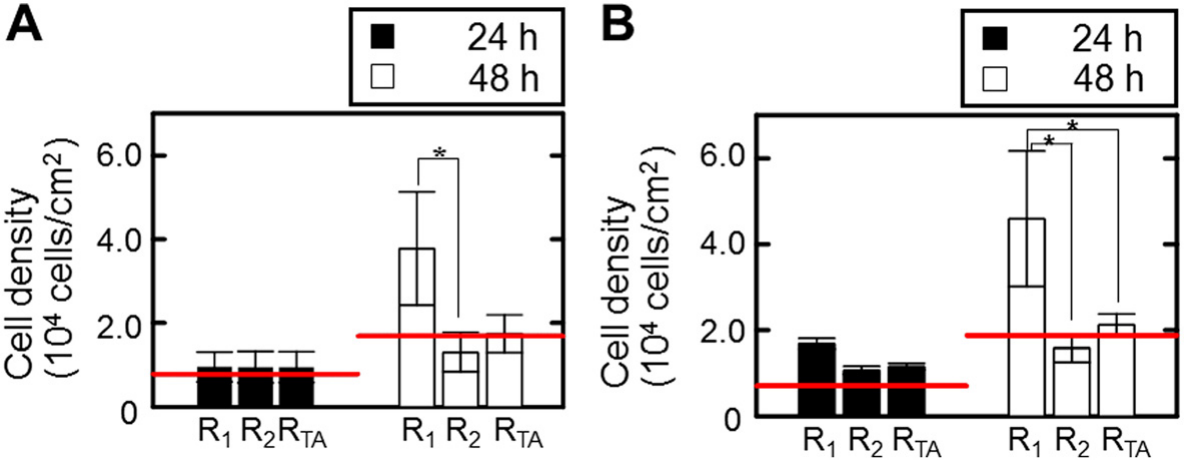
**Vertical spatial growth and distribution of epithelial cells cultured for 48 h on pillar (A) and pit (B) substrates.** The red lines indicate the data determined on the flat unpatterned substrate. Data are measured from individual pillars and pits of 9. The bar shows the standard deviation obtained from triplicate measurements.

The cultures of epithelial cells were performed for 48 h on micropatterned substrates with pillar and pit. As shown in Figure 2, the densities of cells in R_1_ and R_2_ regions on the pillar substrate were respectively 9.6 × 10^3^ and 9.6 × 10^3^ cells/cm^2^ at 24 h, similar to the density in R_TA_. Cell densities were increased for pillar and pit substrates in comparison with those on flat unpatterned substrate (7.1 × 10^3^ cells/cm^2^). At 48 h, the cell densities in R_1_ and R_2_ regions on the pillar substrate were 3.8 × 10^4^ and 1.3 × 10^4^ cells/cm^2^, respectively. These were 4- and 1.4-fold enhancements compared with those flat unpatterned substrate.

The growth ability of cells in the R_1_ and R_2_ regions for the pillar and pit substrates after 48 h is clearly shown in Table 1. Compared with cells on the flat unpatterned substrate, the ratio of adherent cells from 48 h to 24 h (*X*_48_/*X*_24_) in the R_TA_ region of pillar and pit substrates was lower, but significantly different from that in the R_1_ and R_2_ regions. The *X*_48_/*X*_24_ corresponding to the R_1_ region of pillar and pit substrates was 3.9 ± 1.8 and 2.7 ± 1.4, respectively, which was 2.8 and 1.8-fold enhancements as compared with those in the R_2_ region. However, the ratio of dividing cells to the total number of cells (*R*_d_) for 48 h in R_1_ region was similar to that for the R_2_ region, irrespective of the substrate’s topographical feature.

**Table 1:** Growth ability of epithelial cells cultured for 48 h on micropatterned substrates with pillar and pit

**Locational classification of convex or concave in vertical direction**	**Pillar substrate**		**Pit substrate**	
	**Ratio of increased cell number,** ***X***_**48**_**/*X***_**24**_**(-)**	**Ratio of dividing cells,** ***R***_**d**_**(10**^**-2**^**)**	**Ratio of increased cell number,** ***X***_**48**_**/*X***_**24**_**(-)**	**Ratio of dividing cells,** ***R***_**d**_**(10**^**-2**^**)**
R_TA_ region	1.8 ± 0.8	91.3 ± 7.3	1.8 ± 0.5	94.0 ± 3.9
R_1_ region	3.9 ± 1.8	90.9 ± 8.4	2.7 ± 1.4	89.2 ± 8.0
R_2_ region	1.4 ± 1.0	91.4 ± 7.1	1.5 ± 0.4	94.3 ± 5.6
Flat Region	2.1 ± 0.7	84.0 ± 8.4	2.6 ± 0.6	96.1 ± 2.1

Measurements were made in an individual 9 pillars and pits. Data are expressed as means ± standard deviation from triplicate measurements and analyzed using Student’s *t*-test. There were no statistically significant differences among the data sets.

**Figure 3:**
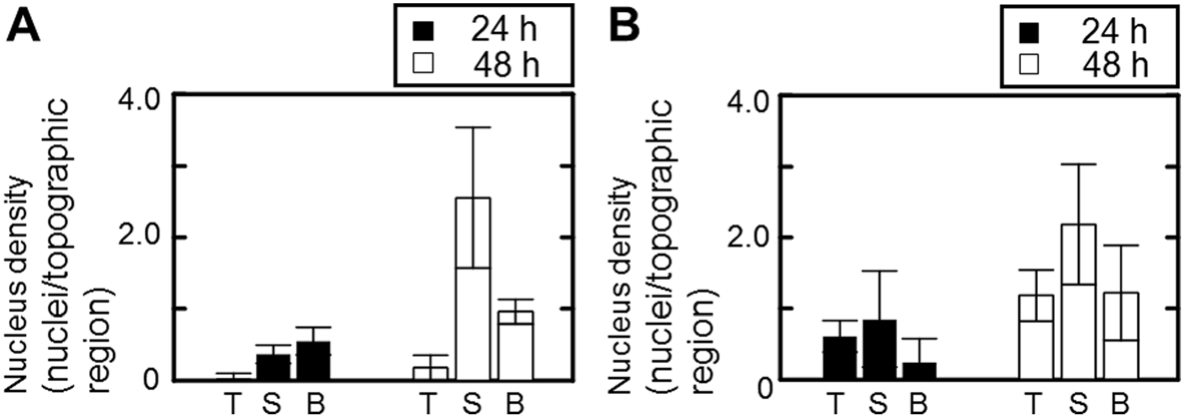
**Horizontal spatial growth and distribution of epithelial cells cultured for 48 h on pillar (A) and pit (B) substrates.** Data are measured from individual pillars and pits of 9. The bar shows the standard deviation obtained from triplicate measurements.

Based on the determined coordinates of nuclei (*x*, *z*), the spatial distribution and nucleus density on the micropatterned substrates occurred on the top surface, sidewall, and/or bottom of the substrate. As shown in Figure 3, cells were predominantly restricted to the sidewalls of the pillar substrate, and to the sidewalls and bottoms of pit substrate. For the pillar substrate, nucleus densities were 0.04, 0.37 and 0.56 nuclei/topographic region at the top, sidewall and bottom, respectively after 24 h. After 48 h, the nucleus density at the sidewall was higher than at the top and bottom. For the sidewall, nucleus density was 2.6 nuclei/topographic region, which was 13.5- and 5.5-fold higher than those at the top and bottom. For the pit substrate, similar trends regarding nucleus density were observed, with 0.61, 0.85 and 0.24 nuclei/topography for the top, sidewall and bottom at 24 h. The densities of nuclei at the sidewall after 48 h were 1.8-fold higher than those at those at the top and the bottom.

### Behavior of epithelial cells on micropatterned substrates

To understand the dynamic behavior of epithelial cells on the micropatterned substrates, we conducted time-lapse observations of representative cells on the pillar and pit substrates as well as the flat unpatterned substrate. As time elapsed, cells exhibited active division and migration, irrespective of topographical feature (Additional file 2: Movie S1, Additional file 3: Movie S2, and Additional file 4: Movie S3). Cells on the pillar substrate migrated towards the sidewall, or stayed at the top of the surface. However, cells on pit substrate tended to move towards the sidewalls and bottom, or confined at the bottoms of the surface. Additionally, cells in the gaps tended to cling to attaching cells at the sidewalls, and to have extended protrusions to the sidewalls. In contrast, cells on the flat unpatterned substrate formed a cluster of cells through division and migration, and rotated persistently in constrained spaces.

### Cytoskeletal formation of epithelial cells on micropatterned substrates

**Figure 4:**
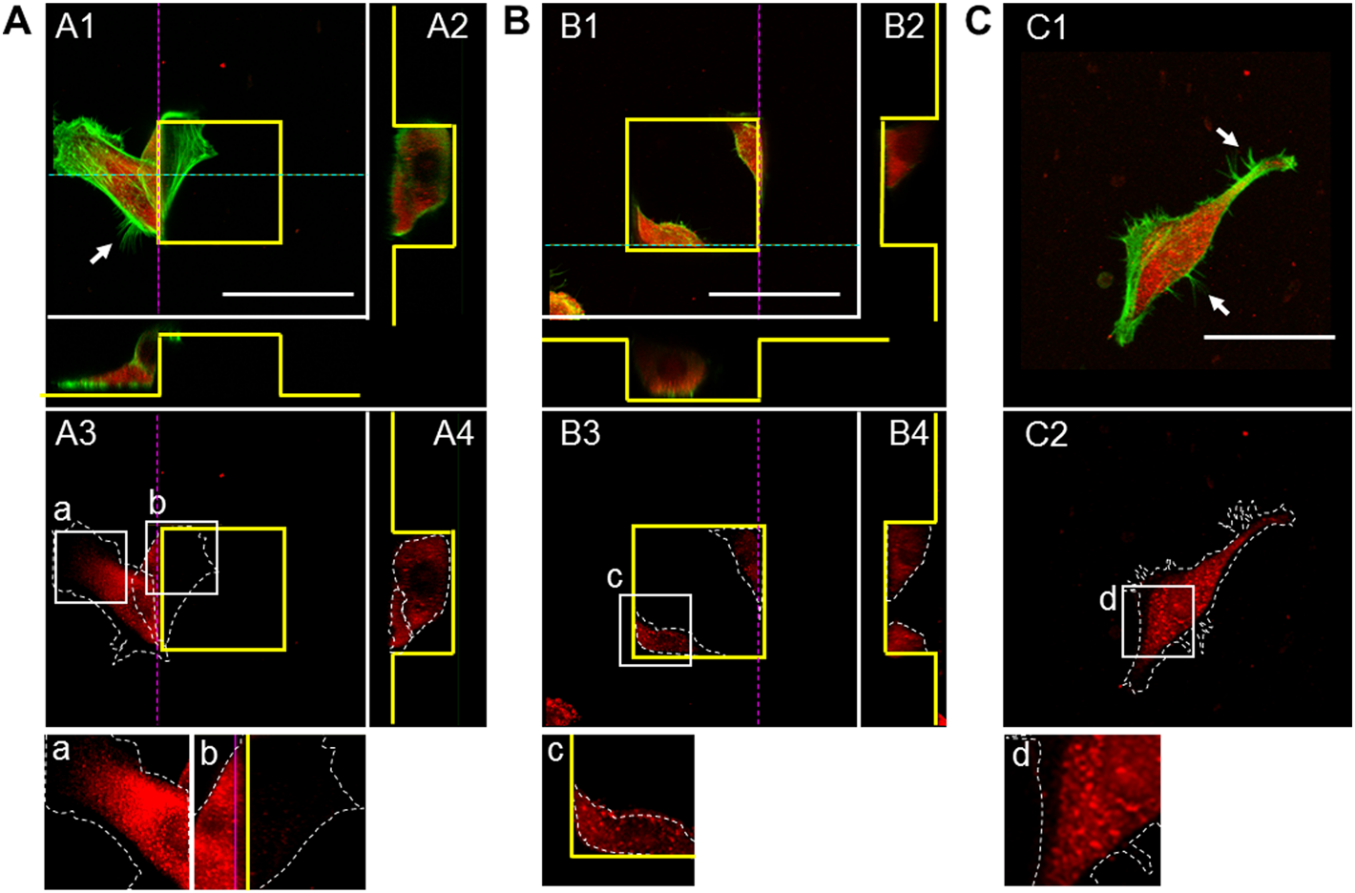
**Immunostaining of vinculin (red) and F-actin (green) for epithelial cells cultured on pillar (A), pit (B) and flat (C) substrates.** The scale bars show 50 μm. The yellow lines outline the pillar or pit features. The white dotted lines indicate the formation of actin stress fibers. Panels A2, A4, B2, and B4 show the cross-sectional views (dotted pink and blue lines) of the images in panels A1, A3, B1, and B3, respectively. Panels a–d show merged enlargements of the corresponding boxed areas in panels A3, B3, and C2. The asterisks indicate the formation of filopodia.

To confirm the cytoskeletal organization and focal contact of epithelial cells cultured on the micropatterned substrates for 48 h, staining of F-actin and vinculin was conducted. For cells on the pillar substrate, stress fibers assembled along straight edges, with extended lamellipodia and filopodia at the sidewalls and convex corners of the structure; this was also seen for cells on flat unpatterned substrate (Figure 4A1 and Figure 4C1). Immature vinculin spots were detected at the cytoplasm and periphery of cells at the sidewalls and convex corners of pillar substrate (Figure 4A3(a) and Figure 4A3(b)). In contrast, the formation of the contractile stress fibers with lamellipodia and filopodia were seldom seen at the concave corners of the pit substrate (Figure 4B1). The formation of the distinct spots of vinculin became pronounced appeared (Figure 4B3(c)).

### Cell-cell contacts of epithelial cells on micropatterned substrates

**Figure 5:**
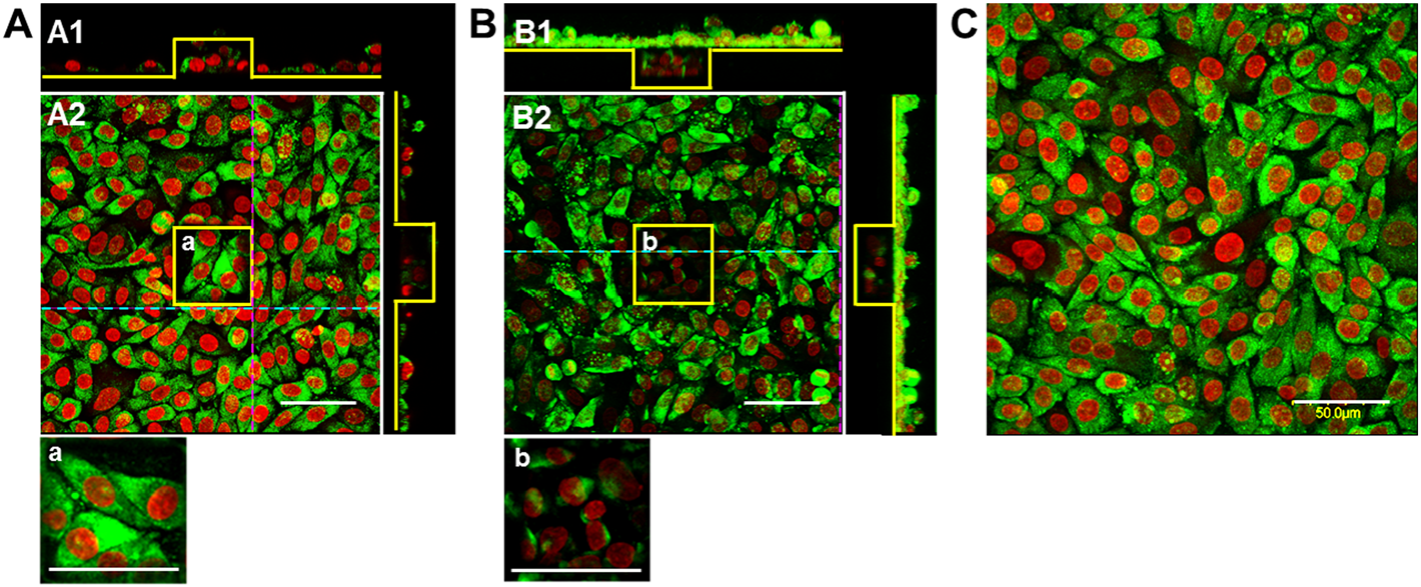
**Immunostaining of E-cadherin (green) for epithelial cells cultured on pillar (A), pit (B) and flat (C) substrates.** Nuclei are stained in red. The scale bars show 50 μm. The yellow lines outline the pillar or pit features. Panels A1 and B1 show the cross-sectional views (dotted pink and blue lines) of the images in panels A2 and B2. Panels a and b show merged enlargements of the corresponding boxed areas in panels A2 and B2.

To investigate contribution of E-cadherin to cell adhesion, the expression of E-cadherin in the cells cultured on the micropatterned substrates with pillar and pit for 144 h was observed. As shown in Figure 5, expression of E-cadherin was observed in almost all cells on the pillar substrate, similarly to those on the flat unpatterned substrate. However, cells on the pit substrate dispersed as confluent monolayers with low levels of E-cadherin expression at the bottom (Figure 5B2(b)).

## Discussion

In this study, we have described cellular responses to 3-D topographical surface features. We compared spatial growth and distribution on two micropatterned substrates with well-defined anisotropic topographical cues. The topographical features of pillar and pit substrates with convex and concave architectures caused heterogeneous spatial growth and distribution in vertical and horizontal directions (Figures 2 and 3). At 24 h, the regional densities and distribution on the pillar substrate was similar to those on pit substrate, regardless of their topographical region. At 48 h, the highest cell densities were observed at the sidewalls of pillar substrate, and at the bottom and sidewalls of pit substrate, respectively. However, there were no significant differences between the topographical regions in the growth ability with number of dividing cells from 24 h to 48 h (Table 1). These results suggest that the topographical features of pillar and pit possibly limit the ability of cells to migrate vertically and horizontally.

**Figure 6:**
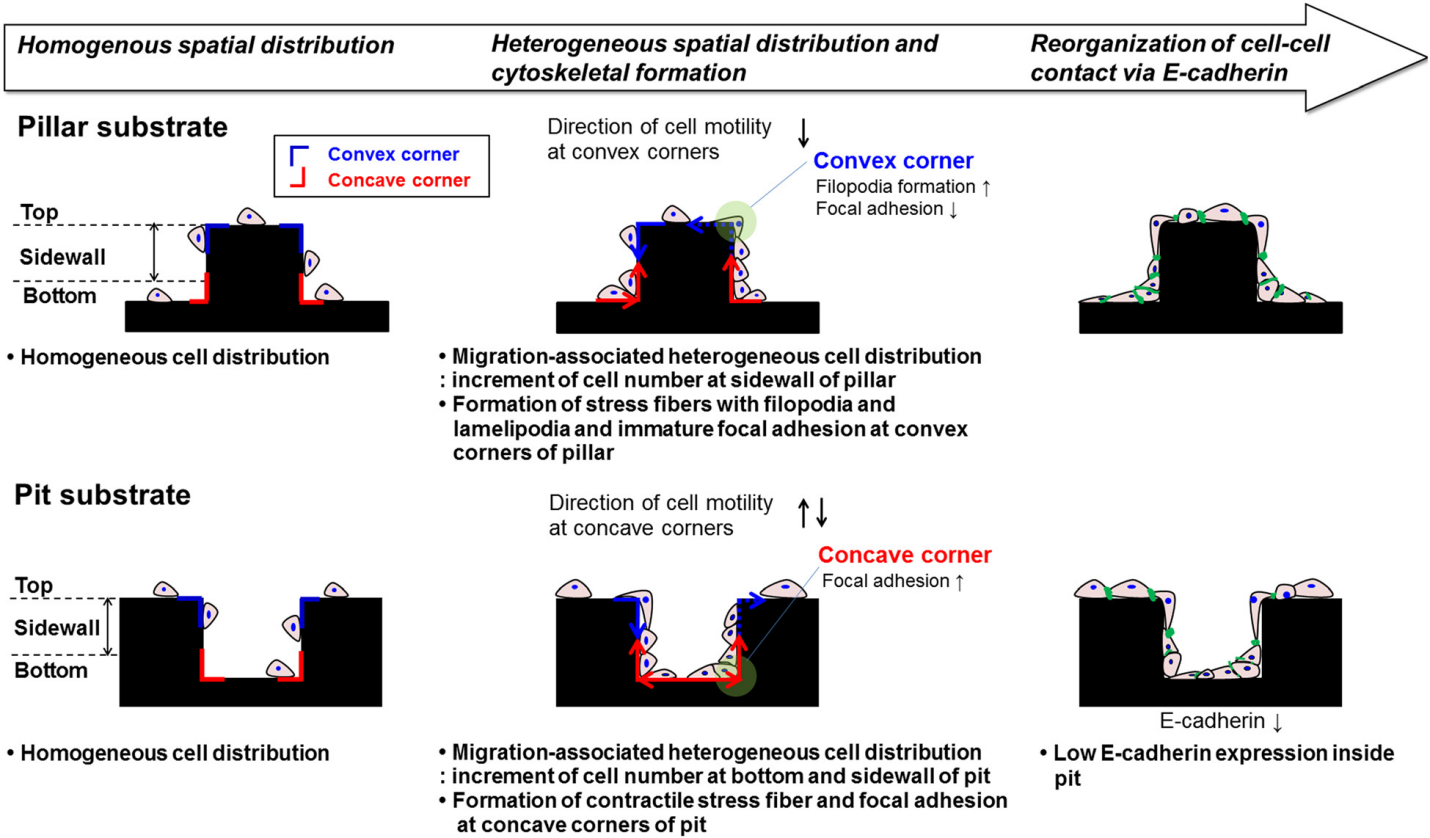
Schematic summarizing effect of micropatterned substrates with pillar and pit on cell behaviors observed in this study.

Surface topography is critical to guide cellular behaviors such as adhesion, spreading and migration [2,8]. Responding to the topographical features, cells protrude their leading edge. The extension of membranes toward the direction of motility, including both lamellipodia and filopodia, brings attachment and thus the traction force to the substrate, resulting in a counter-force on the cell to promote cell migration [1,7]. With these steps of cell migration in mind it follows that surface topography may modulate direction of motility through contact guidance, as the reaction of cells to topographical features leads to cell polarization, lamellipodial and filopodial protrusion, actin bundle alignment, and focal adhesion formation preferentially along these surface features. In the present study, we address the spatial responses of epithelial cells to the micropatterned substrates with pillar and pit. We propose that the heterogeneity of spatial cell distribution, induced by directional migration, is caused by the convex and concave corners of pillar and pit substrates in Figure 6. Cells on pillar substrate responded to convex corners by altering morphology and migrating from the top to the bottom of the surface (Additional file 2: Movie S1), suggesting that they might be predominantly located in sidewalls connecting the top and bottom surfaces. For cells grown on the pit substrate, cells migrated to the sidewalls and bottom of the pit substrate, often bridging corners and attaching to both the bottom and sidewalls. Cells at bottom had an apparent preference for settling at the bottom of the concave cavity, suggesting that they might be sensitive to changes at concave corners (Additional file 3: Movie S2). It indicates that the convex and concave corners possibly limit the ability of cells to migrate vertically and horizontally. It is therefore possible to selectively influence either cell adhesion or morphology via surface topography. These observations indicate that orientation, migration, and morphology of the cells appeared to be governed by topographical features of the convex and concave corners of the pillar and pit substrates. It is not clear, however, whether morphological polarity of the cell itself at convex and concave corners of pillar and pit substrates can determine the direction of movement. The different patterns of movement during migration take place at the convex and concave corners of pillar and pit substrates, resulting in heterogeneity of spatial cell distribution.

Although a detailed mechanism for the transduction of such topographic signals is unclear, one possibility is that mechanical forces are transmitted through integrins. This might cause an associated sensing protein on the cytoplasmic side to alter its conformation and enzyme-substrate activities [3,4]. Consequently, cytoskeletal organization and adhesion to a substrate alters the way in which cells sense and respond to their microenvironment, thereby affecting cell-cell contact [18]. The cytoskeleton of a moving cell’s protrusion is the property that determines such reactions to topography. We found that the response of cells upon encountering a topographical feature could be predicted. Convex and concave topographies have attracted significant attention with regards to providing insights into the mechanisms of cell migration [2]. Spatial organization and dimensions of these micropatterns have been shown to affect the migration of cells [2,7]. Ghibaudo *et al.* investigated the role of substrate topography in cell adhesion and migration [18]. The topographical features of convex substrate induced changes in cellular morphology that were caused by alterations in the cytoskeleton in response to focal adhesion formation. Focal adhesions are known to serve as membrane sensing entities that control local and global adhesion mediated by Rho family GTPases signaling [19-23]. Rac1 and cadherin appear to be the major players in the maintenance of epithelial cell morphology [18,19]. Previous studies have demonstrated that this switching mechanism occurs in cells moving from 2-D to 3-D environments because of lower Rac1 activity in 3-D cell cultures [18]. Thus, the location and patterns of adhesive sites imposed by substrate topography most probably drive the direction of cell migration by modulating RhoA and Rac1 signaling pathways, and thus, cell polarity, adhesion and traction forces [18]. Furthermore, Rac1 signaling has emerged as a key regulator of E-cadherin-mediated cell–cell adhesion [23]. In this study, we found that variations in the spatial distribution and cytoskeletal structure of cells on the micropatterned substrates were dependent upon altered migration. Cytoskeletal staining for F-actin and vinculin showed that cells cultured on pillar substrate had stress fibers with extended filopodia and immature focal contacts at the sidewalls and bottom corners, similar to those seen on the flat unpatterned substrate (Figure 4A and Figure 4C). However, cells at the sidewalls and concave corners of the pit substrate had contractile stress fibers and more stable focal contacts than convex corners of the pillar substrate (Figure 4A and Figure 4B). Overall there were more developed filopodia and immature adhesions at the convex corners of pillar substrate compared with concave corners of pit substrate, possibly accounting for increased migration. This suggests that cells followed the convex corners and migrated toward the sidewalls of pillar substrate. Based on our observations it is plausible that the location for focal contacts on the micropatterned substrate is specified by spatial restrictions where cells can favorably form contact points with the surface. In addition to cytoskeletal formation, E-cadherin expression was seen for cells attached to the top of the pillar substrate. This expression was reduced in cells toward the bottom of the pit substrate (Figure 5b). When stable cell adhesion occurs inside a concave structure, stress in the actin cytoskeleton is induced. This disrupts the cytoskeleton during persistent migration, limiting the intracellular pathways responsible for cell-cell contact formation. We propose that epithelial phenotypes at the convex and concave corners of micropatterned substrates are regulated by the actin cytoskeletal architecture through cell–cell and cell–substrate interactions. These, in turn, elicit different contractile forces and levels of adhesion to stimulate cell migration. Taken together, our results demonstrate that anisotropic topographical features are important factors that affect spatial growth and distribution. Heterogeneity was caused by differences in migratory behavior at the convex and concave corners of pillar and pit substrates.

## Conclusion

Our study supports the notion that the evaluation of spatial distribution in culture of epithelial cells can help in understanding the proliferation and migration potentials of cells grown on the pillar and pit substrates. Variations in orientation, migration, and morphology of cells were dependent upon substrate topography. We found that this altered migration could be attributed to spatial reorganization of the actin cytoskeleton, and formation of focal adhesions at various points along the convex and concave corners of pillar and pit substrates. Cells cultured on the pillar substrate had stress fibers with extended filopodia and immature focal contacts at the sidewalls and convex corners of substrate. Cells at the sidewalls and concave corners of pit substrate had contractile stress fibers, and formed more distinct focal contacts than cells on the pillar substrate. The convex and concave corners of pillar and pit substrates possibly created patterns of mechanical forces that modulate the level and direction of intracellular forces. The anisotropic topographical features of micropatterned substrates affected the spatial growth and distribution, thereby changing cell-cell and cell-surface interactions. Our investigations could lead to an effective approach for the design of the materials to control cell migration, and in the design of implants for tissue engineering applications.

## Competing interests

The authors declare that they have no competing interests.

## Authors’ contributions

Conceived and designed the experiments: MHK, YS, MT, MK. Performed the experiments: MHK, YS. Analyzed and interpreted the data: MHK, YS, MT, MK. Wrote the paper: MHK, MT, MK. All authors read and approved the final manuscript.

## Supplementary Material

Additional file 1: Figure S1 Substrate topography. (A) Schematic showing two micropatterned substrates with pillar and pit. (B) Scanning electron microscopy images of micropatterned substrates with pillar (B1) and pit (B2). Click here for file

Additional file 2 **Movie S1.** Dynamic behavior of epithelial cells cultured on pillar substrate. Click here for file

Additional file 3 **Movie S2.** Dynamic behavior of epithelial cells cultured on pit substrate. Click here for file

Additional file 4 **Movie S3.** Dynamic behavior of epithelial cells cultured on flat substrate. Click here for file
