# Supplementary figures and images for: Influence of surface topography on the human epithelial cell response to micropatterned substrates with convex and concave architectures
Source: J Biol Eng. 2014 Jun 19;8:13. doi: 10.1186/1754-1611-8-13 (PMC4084502; doi:10.1186/1754-1611-8-13)

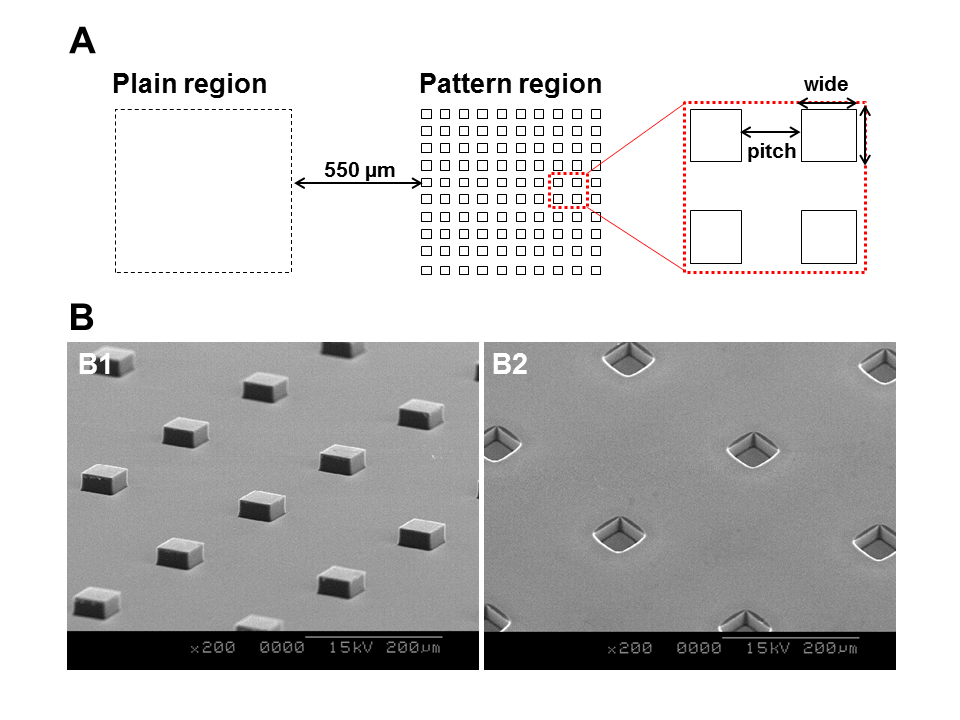

Supplement: Additional file 1: Figure S1 — Substrate topography. (A) Schematic showing two micropatterned substrates with pillar and pit. (B) Scanning electron microscopy images of micropatterned substrates with pillar (B1) and pit (B2). [file 1754-1611-8-13-S1.tiff]
